# Geopolitical impacts on the description of new terrestrial mollusc species

**DOI:** 10.1098/rspb.2025.1428

**Published:** 2025-09-17

**Authors:** Evandro C. T. Abreu, Edson Lourenço da Silva, Mario R. Moura

**Affiliations:** ^1^ Programa de Pós-Graduação em Biodiversidade, Universidade Federal da Paraíba, Areia, Paraiba, Brazil; ^2^ Laboratório de Biologia, Instituto Federal de Educação Ciência e Tecnologia do Piauí - Campus Picos, Picos, Brazil; ^3^ Departamento de Sistemática e Ecologia, Universidade Federal da Paraiba, João Pessoa, Paraiba, Brazil; ^4^ Departamento de Biologia Animal, Universidade Estadual de Campinas, Campinas, Sao Paulo, Brazil

**Keywords:** alpha taxonomy, biodiversity shortfall, knowledge gap, neocolonial practices, parachute science, zoology

## Abstract

Despite 2.3 million described species, biodiversity knowledge remains incomplete and unevenly distributed. We analysed 20 years of terrestrial mollusc descriptions, categorizing collaborations as domestic (authored by resident researchers only), parachute (foreign researchers only) or mixed (joint collaboration) to assess links between discovery practices, socioeconomic factors, methodological rigour (number of type specimens, morphometric characters, evidence lines and publication length) and accessibility to analytical tools and resources (internal anatomy, molecular biology and taxonomic revisions). Global South harboured 88.6% of parachute discoveries, with resident researchers involved in 33.3% of total descriptions. Exclusionary practices resulted in lower first-authorship rates for Global South researchers and frequent omission of fieldwork personnel. Collaborations were asymmetrical: nearly 90% of Global South-led studies included Northern researchers, but only 8% of Northern-led studies included Southern partners. Economic power correlated with absolute and relative parachute discovery outputs, while mixed collaborations improved Global South access to analytical tools—although their descriptions remained less comprehensive. Parachute discoveries in the Global South showed lower methodological rigour, underscoring the cost of excluding local expertise. Taxonomic revisions, which were 89% led by the Global North, further reflected resource disparities. Equitable international collaborations prioritizing local capacity-building are crucial for achieving global biodiversity knowledge and advancing conservation goals.

## 1. Introduction

Approximately 2.3 million species have been described worldwide [1]. Although substantial, this figure represents only a fraction of Earth’s species, with most still unknown to science [2–4]. This knowledge gap has been recognized in international agreements, such as the Convention on Biological Diversity [5], due to its potential to hinder our understanding of biodiversity patterns and processes and affect conservation planning [6–8]. Despite being a global issue, this gap is not uniformly distributed across the planet [9,10], and may be influenced by socioeconomic and cultural disparities that also impact contemporary science [11]. Countries in the Global North, with greater economic development, generally have better-documented biodiversity than those in the Global South, which harbours the most poorly studied or even unknown species [9,10,12–14]. This biodiversity knowledge paradox, better sampling in the Global North and higher biodiversity in the Global South [14,15], may encourage researchers from wealthier nations to conduct studies in the Global South.

While international efforts to catalogue planetary biodiversity are widely needed [16], multilateral collaborations must promote academic practices that foster technology transfer and recognize the intellectual contribution of resident researchers [17]. Such an approach would more effectively catalyse knowledge production on biodiversity in the Global South. Recent studies show an increase in the number of authors, institutions and countries involved in taxonomic research over time [18,19]. This growing internationalization of taxonomy reflects, in part, the establishment of new techniques and analytical tools for species identification and delimitation, such as DNA barcoding [20,21] and micro-computed tomography (CT) [22], fostering cooperation between researchers from different institutions [23]. Nevertheless, taxonomy's internationalization can lead to parachute science, where scientists from foreign, higher-income institutions describe species collected in another, typically lower-income, country, without effective communication or engagement with local researchers, as recently shown in palaeontology [24].

Parachute science is not the sole evidence of scientific colonialism. More pervasive neocolonial practices systematically marginalize researchers residing in the Global South, reinforcing the historical dominance of institutions from wealthy countries [25,26]. We note that researcher residency (affiliation), rather than nationality, may more accurately reflect systemic disparities in scientific participation [27]. For instance, a Global South researcher based in the Global North gains access to advanced infrastructure and funding opportunities, whereas a Global North researcher based in the Global South often operates under local constraints [16]. That said, neocolonial practices can result in the underrepresentation of Global South resident researchers in leadership roles (e.g. first authorship) or their limited presence in regionally confined collaborations, where only Global North researchers are invited to collaborate without reciprocity [28]. These practices may hinder the emergence of new research centres and exacerbate global scientific inequalities [29]. The lack of academic inclusion can delay scientific development and the subsequent reduction of biodiversity knowledge gaps in megadiverse regions, compromising the achievement of goals established under the Post-2020 Global Biodiversity Framework.

Scientific colonialism can extend beyond authorship disparities to deeper consequences for accessibility of research tools and resources. While recent studies have examined colonialist patterns in research effort and publication counts [24,27,30], their impacts on the ‘methodological rigour’ of taxonomic descriptions remain virtually unexplored. This methodological rigour is not captured by conventional citation-based metrics [31,32], being reflected in proxies like (i) number of type specimens (the primary reference material used to described a new species), (ii) number of morphometric characters, (iii) page length, and the (iv) quantity of evidence lines or number of techniques adopted in formal description (e.g. morphology, molecular biology, internal anatomy, among others). These proxies—documented in several taxa, including mammals [33], birds [34], reptiles [35], helminths [36], fossils [37], among others [38]—can reflect systemic inequities in access to field, laboratory and analytical resources [35,36,39]. Such disparities likely compound traditional metrics of exclusion (e.g. authorship exclusion and marginalization [24,29]), further disadvantaging biodiversity research in megadiverse regions.

Here, we examine 20 years of species descriptions to assess how neocolonial academic practices influence species discovery of new species. Focusing on post-2000 descriptions minimizes inconsistencies from historical variations in taxonomic standards, ensuring comparable scientific and socioeconomic trends. We ask three major questions: (1) do species descriptions from the Global South show greater exclusion and marginalization of resident researchers compared to those from the Global North? (2) Is the per-country incidence of exclusionary practices associated with the economic power of country’s authors? (3) Does the use of advanced tools and resources (underlying the comprehensiveness of taxonomic descriptions) differ between discoveries from the Global South and North? Under neocolonial dynamics, we expect exclusionary practices disproportionately affecting resident researchers in the Global South, with wealthier nations from the Global North exhibiting both higher rates of parachute science and greater accessibility to resources and tools commonly applied to species description. We use molluscs as a model system, focusing on terrestrial species to avoid confounding factors from marine species sampled in international waters [40]. Terrestrial molluscs are the most diverse animal group after arthropods, with representatives on all continents except Antarctica [41]. This group has shown an intense history of species discoveries in recent decades [42], with the 90K known species comprising about 45% of the estimated global mollusc richness [43]. Thus, ongoing mollusc discoveries offer opportunities to incorporate more inclusive academic practices and reshape current paradigms to accelerate species documentation. Nonetheless, our taxon-independent metrics of exclusionary practices and proxies of description comprehensiveness [38], in concert with the global scope, help yield broader implications for equitable taxonomic practices worldwide.

## 2. Material and methods

### (a) Species data

We searched the literature for 3633 descriptions of extant terrestrial mollusc species published from 2003 to 2022, as informed in the MolluscaBase platform [44], all of which belong to the class Gastropoda. These contemporary descriptions now routinely integrate molecular data, high-resolution imaging and comprehensive morphological analyses, unlike older works that relied on limited specimens and superficial traits. We sourced publications through SciELO, Pensoft, Elsevier, Google Scholar and ResearchGate, and directly contacted authors for unavailable descriptions. Our final dataset comprised formal descriptions for 3272 terrestrial mollusc species (90% of total), representing 94.8% of families and all orders within the class.

For each species, we obtained the type-locality country from geographical coordinates informed or through gazetteers (e.g. https://earth.google.com) when only place names (e.g. cities or protected areas) were provided. By comparing the nationality of the type locality with the authors’ affiliated countries, we classified species descriptions into three categories of research collaboration: (i) domestic (authored by resident researchers only), (ii) parachute (authored by foreign researchers only), or (iii) mixed (including both resident and foreign researchers). To measure marginalization of resident researchers, we recorded whether the first author was a resident researcher in the species’ country of origin. When multiple affiliations were present, only the first one was considered. This conservative approach aims to minimize the inclusion of ‘non-legitimate’ multiple affiliations where at least one of the affiliations does not reflect a substantial contribution [45]. Additionally, we assessed whether collectors of type specimens authored species descriptions as a measure of inclusivity toward fieldwork personnel, rather than marginalization. While we assume collectors to reflect the availability of local expertise, we acknowledge that foreign collectors (not tracked here) may also go unrecognized.

We measured species description comprehensiveness using four proxies: (i) number of type specimens, as an indicative of field effort and access to diverse collections; (ii) count of absolute morphometric characters (excluding ratios), indicating the depth of morphological analysis; (iii) page length, used as an indicator for overall descriptive detail, measured by dividing each page into quadrants and counting only those within Methods/Results sections [35]. For work comprising multi-species descriptions, we divided page counts by the number of species described. The fourth proxy, (iv) number of evidence lines, was scored from 1 to 5 based on the inclusion of morphometry, molecular analysis, morphology, internal anatomy and coloration pattern in the formal descriptions, and demonstrates the integration of multiple analytical tools and potentially greater resource investment. These proxies showed low multicollinearity (variance inflation factor, VIF < 1.2) and limited pairwise correlations (Kendall’s τ coefficient range = 0.05–0.36; electronic supplementary material, table S1), indicating they capture relatively distinct aspects of description comprehensiveness.

To complement our investigation on the accessibility of resident researchers to specific analytical tools and resources, we recorded whether descriptions derived from taxonomic revision, defined as studies explicitly using terms like ‘revision’ or similar in their title, abstract, keywords or main text. Revisionary studies demand substantial resources to visit several collections and examine extensive reference material [46,47], making their occurrence, alongside costly methodologies like internal anatomy and molecular analysis [38], a meaningful indicator of access to analytical tools and resources. Similarly to the continuous proxies informed above, discrete variables considered here (i.e. taxonomic review, molecular biology, internal anatomy, morphology and coloration pattern) showed low redundancy (Phi’s φ coefficient range = 0.00–0.22 for all pairwise correlations, except incidence of internal anatomy and molecular biology, φ = 0.49, generalized-VIF < 1.4; electronic supplementary material, table S2). All database operations were performed using the *dplyr* [48] and *data.table* [49] packages in the R software v. 4.3.3 [50].

### (b) Country data

We gathered data on type-locality country and countries mentioned in the authors’ affiliations to compile a list of nations involved in terrestrial mollusc discovery between 2003 and 2022. For each country, we employed the geopolitical classification adopted by the United Nations [51] to obtain the (i) ISO-alpha3 code, a three-character abbreviation representing each country’s name; (ii) geopolitical location of the country in the Global North or Global South; and (iii) geopolitical region to which the country belongs (Sub-Saharan Africa; Latin America and the Caribbean; North America; Australia and New Zealand; Central, Eastern and Southern Asia; Europe; Near East and Northern Africa; Southeast Asia and Pacific Islands). Japan, South Korea, Israel and French Guiana are classified as part of the Global North, although they are typically included in geopolitical regions dominated by countries in the Global South. For greater geographic detail, we removed the Russian Federation from Europe to represent it as an independent geopolitical region. In total, 72 countries were identified at this stage.

Using the three categories of research collaboration for species description (domestic, mixed, parachute), we calculated four country-level metrics of discovery output: number of (i) total discoveries (descriptions in domestic or foreign territory); (ii) foreign discoveries (descriptions in foreign territory only, with or without resident researchers); (iii) parachute discoveries (descriptions in foreign territory excluding resident researchers); and the (iv) proportion of parachute discoveries with respect to total discoveries. We also used each country’s ISO-alpha3 code to extract the per capita gross domestic product (GDP), adjusted for purchasing power parity (PPP), using the *wb_data* function from the *wbstats* package [52]. This indicator is provided annually via the World Bank Open Data platform (https://www.worldbank.org). It measures the average production of goods and services per person in a country, considering differences in living costs and purchasing power across countries. In this study, we averaged the per country GDP–PPP across the last two decades. In a complementary manner, we also assessed per-country investments in research and development expenditure as a percentage of GDP.

### (c) Data analysis

To characterize neocolonial practices, we categorized species descriptions based on the geopolitical location of the type locality (distinguishing between those recorded in the Global North and the Global South) and the research collaboration type (domestic, mixed, of parachute discovery). We used the chi-square test to examine whether geopolitical location and research collaboration influenced the proportion of original descriptions that (i) included any collector of type specimens, (ii) employed internal anatomy, (iii) used molecular biology techniques, and (iv) resulted from taxonomic revisions. For continuous variables related to the comprehensiveness of taxonomic descriptions, we applied the Kruskal–Wallis (K-W) test to assess whether (v) the type-series size, (vii) number of morphometric characters observed, (viii) description page length, and (v) number of evidence lines exhibited different medians across geopolitical location and research collaboration types. Where appropriate, we log_10_ transformed variables related to the taxonomic comprehensiveness to reduce variable skewness and minimize the influence of outliers. We adjusted the *p*-value tests using the Bonferroni method to account for multiple comparisons. All analyses and data visualizations were conducted in R v. 4.3.3 [50], using the *ggplot2* [53]*, ggpubr* [54] and *stats* [50] packages.

## 3. Results

**Figure 1.**
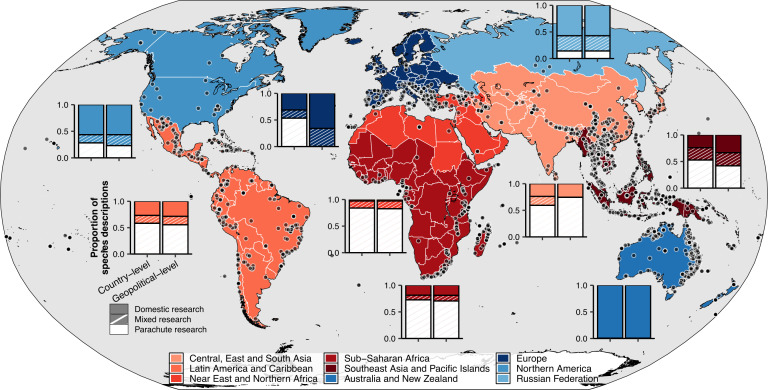
Proportion of species discoveries by type of research collaboration. Each point represents the type locality of terrestrial mollusc species described between 2003 and 2022. Species descriptions were categorized into three types of research collaboration: domestic (authored only by resident researchers), parachute (authored only by foreign researchers) or mixed (collaboration between resident and foreign researchers). Two political levels were used to quantify the proportion of species descriptions: country and geopolitical region.

We obtained information on 3272 terrestrial gastropod species described between 2003 and 2022. Of these, 2216 (68%) were described in 78 countries across the Global South, while 1056 species (32% of the total) were found in 26 countries in the Global North (figure 1). Species were described in all geopolitical regions, with Southeast Asia and the Pacific Islands accounting for the largest share at 32.3% (1057 species). Australia and New Zealand contributed 21.4% (700 species), Sub-Saharan Africa 12.3% (341 species), Central, Eastern and Southern Asia 10.6% (294 species), Latin America and the Caribbean 10.6% (348 species) and the Near East and Northern Africa 2.3% (75 species). North America and the Russian Federation contributed only 1.19% (39 species) and<0.2% (seven species), respectively.

Although most species were described in the Global South, researchers based there contributed to only 33.3% (*n* = 1090) of descriptions. In contrast, researchers based in the Global North contributed to 79.3% (*n* = 2595) of all descriptions. When comparing descriptions between these two geopolitical macroregions, 51.4% of species described in the Global South excluded researchers from this macroregion, whereas only 0.6% of species described in the Global North lacked participation from researchers based there. Exclusively domestic research accounted for 40.4% (*n* = 1321) of species descriptions, while mixed research efforts contributed to 14% (*n* = 458). The remaining 1493 (45.6%) species descriptions were parachute discoveries (i.e. authored by foreign researchers only). Notably, 88.6% (*n* = 1323) of all parachute discoveries occurred in the Global South.

**Figure 2.**
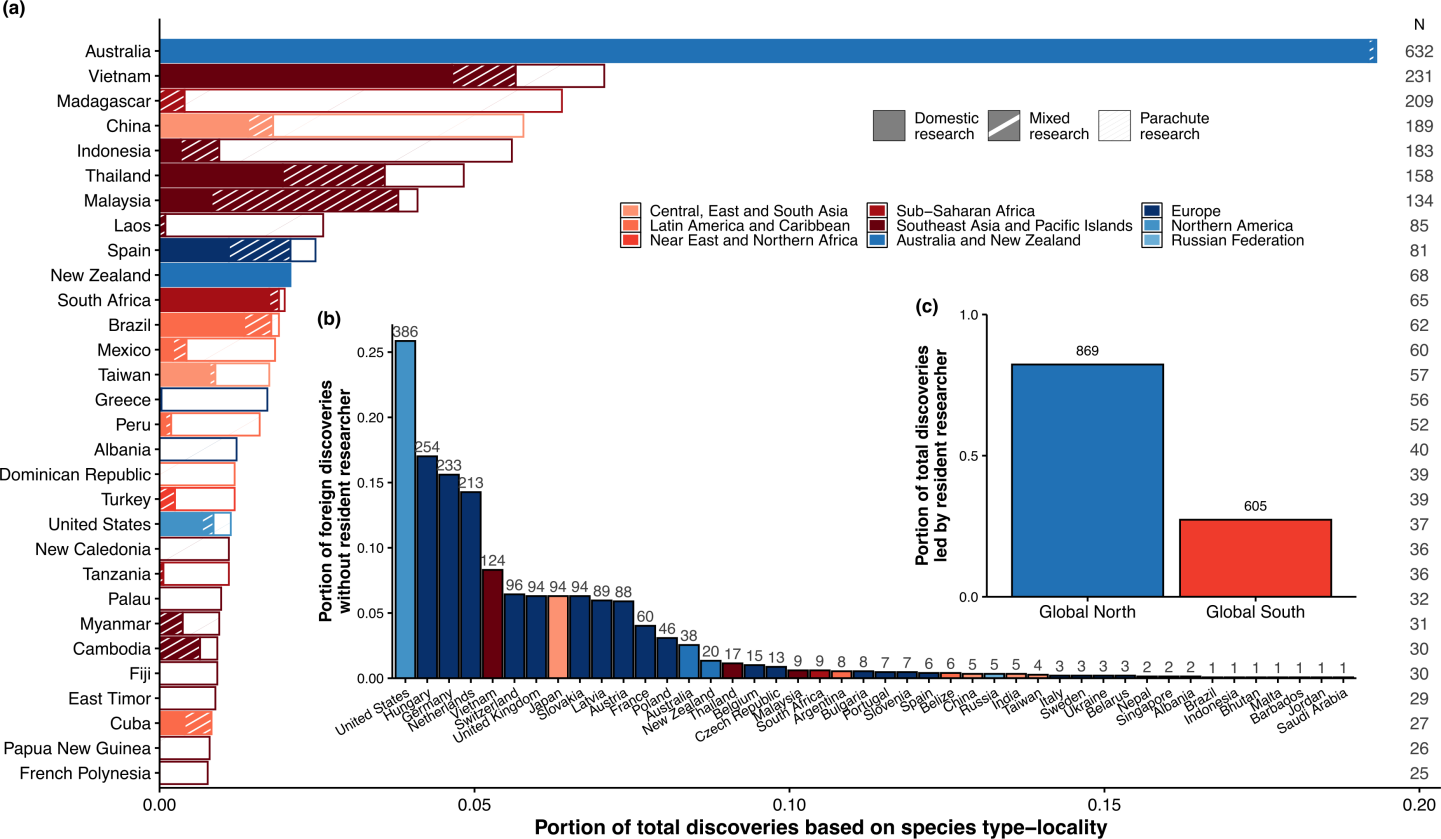
Top 30 countries with the highest number of species descriptions within their territory. (a) Proportion of total species by country according to research collaboration type (domestic, mixed or parachute). Bar colours correspond to the geopolitical regions shown in figure 1. Numbers on the right (*n*) indicate the total number of discoveries within each country’s territory. (b) Countries ranked by their share of total parachute discoveries, with the total number of parachute discoveries shown at the top of each bar. Note that parachute discoveries may involve contributions from multiple countries. (c) Proportion of species descriptions led by resident researchers between the Global North and Global South. See electronic supplementary material, figure S3 for leadership patterns at the country level.

Among the top 30 countries with the highest number of species descriptions within their territories, Australia ranked first, with 632 discoveries (figure 2a). Of the 51 countries conducting species descriptions in foreign territories, 44 excluded resident researchers, with parachute research serving as the primary mode of foreign study for 35 countries (68.6% of those researching abroad; electronic supplementary material, figure S2). The United States accounted for 25.8% of all parachute discoveries, followed by Hungary (17%) and Germany (15.6%, figure 2b). We found disparities in taxonomic leadership, with resident researchers serving as first authors for only 27.3% of Global South descriptions, compared to 82.3% in the Global North (χ² = 871.4, d.f. = 1, *p* < 0.001, figure 2c). While resident researchers led all descriptions in countries like Australia and New Zealand, foreign researchers led all descriptions in several Global South countries (electronic supplementary material, figure S3). This marginalization is further reflected in asymmetrical academic collaborations. While 89.7% of internationally co-authored descriptions (i.e. mixed research) from the Global South involved Global North collaborators, only 8% of such publications from the Global North included Global South collaborators. In other words, South–South partnerships represented just 10.3% of mixed collaborations in the Global South, whereas North–North partnerships accounted for compared to 91.9% of mixed research in the Global North.

**Figure 3.**
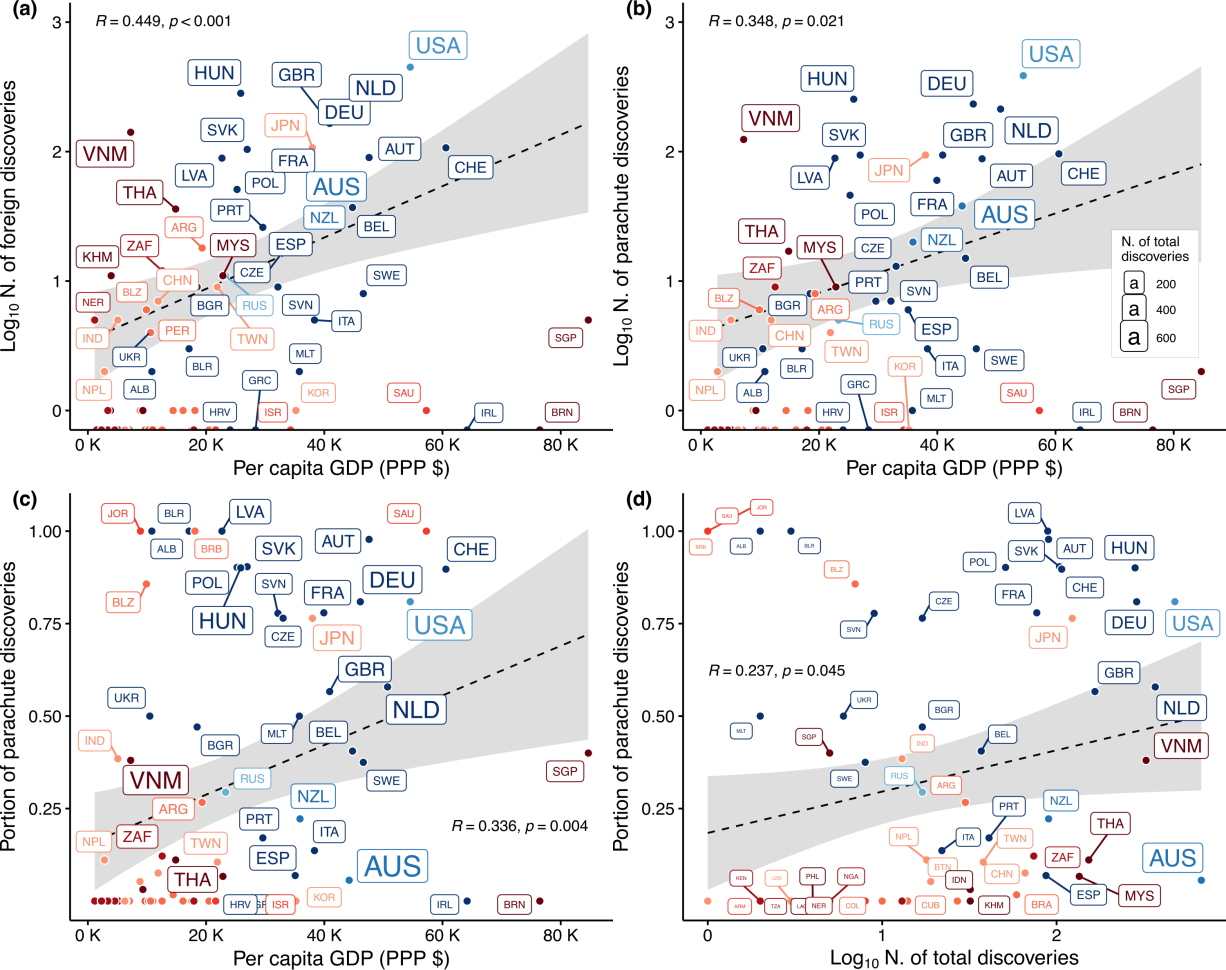
Influence of GDP per capita on species discovery output of foreign and parachute research. Effect of GDP per capita adjusted for PPP on the total number of (a) foreign discoveries, (b) parachute discoveries and (c) proportion of total discoveries abroad that occurred without the involvement of resident researchers. (d) Relationship between absolute discovery output and relative parachute discoveries. Countries are represented by their respective ISO-alpha3 codes, with font size proportional to the total number of discoveries. Text colours follow the geopolitical regions depicted in figure 1. R indicates Pearson’s correlation coefficient and the corresponding *p*‐value.

Across all countries participating in species discovery, we found that per capita GDP–PPP was positively correlated with counts of both foreign (Pearson’s *r* = 0.449, *p* < 0.001, figure 3a) and parachute discovery (*r* = 0.348, *p* = 0.021, figure 3b) and the proportion of species described without resident researchers involvement (*r* = 0.336, *p* = 0.004, figure 3c). These findings remained consistent after excluding countries with exclusively domestic discoveries (electronic supplementary material, figure S4) and when accounting for national R&D investment (electronic supplementary material, figures S5 and S6). However, the positive association between the proportion of parachute discoveries and a country’s total discovery output (*r* = 0.237, *p* = 0.045, figure 3d) was not supported in these sensitivity analyses (electronic supplementary material, figures S4–S6).

**Figure 4.**
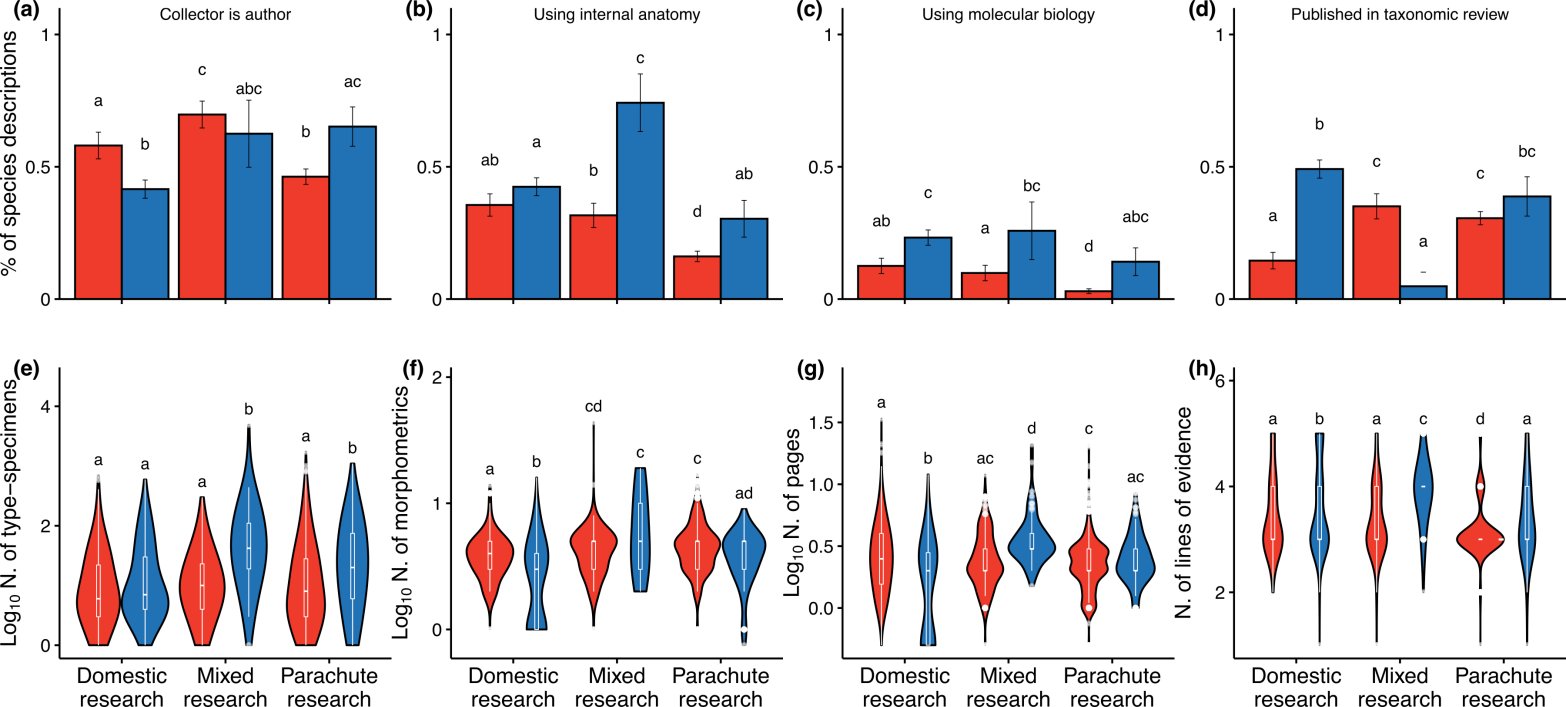
Characterization of discoveries based on participation of fieldwork personnel, access to analytical tools and description comprehensiveness metrics. Differences between factors associated with species descriptions from the Global North (blue) and South (red) across categories of research collaboration (domestic, mixed or parachute research). Proportion of species descriptions (a) authored by at least one type-specimen collector; using techniques of (b) internal anatomy or (c) molecular biology; and resulting from (d) taxonomic revisions. Number of (e) type specimens (log_10_), (f) morphometric characters, (g) pages (log_10_) and (h) lines of evidence used in species descriptions. The lowercase letters refer to pairwise multiple comparisons for proportion (a–d) and continuous (e-h) metrics with Bonferroni corrected *p*-values. See electronic supplementary material, table S3 for summary statistics of each variable.

Type-specimen collectors authored 52.7% of descriptions from the Global South compared to 46.4% in the Global North (electronic supplementary material, figure S7), with these values strongly varying by research type (figure 4). Discoveries based on domestic research in the Global South showed higher collector authorship rates, while the opposite pattern emerged in parachute research, where collectors in the Global North were more frequently credited (χ² = 105.0, d.f. = 5, Bonferroni *p* < 0.001, figure 4a), indicating geopolitical disparities in fieldwork recognition. These disparities extended to analytical approaches. Global North descriptions employed internal anatomy (χ² = 249.3, d.f. = 5, Bonferroni *p* < 0.001, figure 4b) and molecular biology (χ² = 223.4, d.f. = 5, Bonferroni *p* < 0.001, figure 4c) more frequently, particularly in mixed and parachute research. Similarly, taxonomic review was more common in Global North domestic research, though mixed and parachute collaborations increased its use in Global South discoveries (χ² = 199.9, d.f. = 5, Bonferroni *p* < 0.001, figure 4d).

Our analysis reveals larger type-series size in Global North descriptions across non-domestic research (K-W χ² = 85.1, d.f. = 5, *p* < 0.001, figure 4e). Morphometric analyses used more characters in description derived from mixed research (K-W χ² = 396.8, d.f. = 5, *p* < 0.001, figure 4e), despite comparable values between Global South and North macroregions. We also observed the greater comprehensiveness of Global North descriptions, as measured by lengthier descriptions (K-W χ² = 182.7, d.f. = 5, *p* < 0.001, figure 4g) and more evidence lines (K-W χ² = 271.5, d.f. = 5, *p* < 0.001, figure 4h), particularly for species discoveries based in mixed and parachute research. Parachute discoveries included more authors if performed in the Global North rather than the Global South (K-W χ² = 552.1, d.f. = 5, *p* < 0.001, electronic supplementary material, figure S8).

## 4. Discussion

The Global South harbours most of the planet’s biodiversity yet faces lower sampling rates [55,56]. At the same time, neocolonial practices exacerbate inequalities in global science [29]. Our findings reveal stark geopolitical disparities in terrestrial mollusc taxonomy, with severe exclusionary practices towards resident researchers in the Global South that mirror trends in other fields [29,30,57]. While three-quarters of new species were discovered in the Global South, over half of resident researchers were excluded from formal descriptions (figure 1), deepening the biodiversity paradox [9,15]. Compared to researchers in the Global South, those in the Global North have three times higher odds of leading description works (figure 2) and nine times greater odds of engaging in species discovery studies derived from international collaboration, underscoring systemic exclusion and unreciprocated partnerships. Exclusion correlates with national economic power, but not necessarily with local research capacity (figure 3). While international collaborations improve access of Global South resident researchers to analytical tools and resources (figure 4), their taxonomic descriptions remain less comprehensive than those from the Global North.

The dominance of Global North researchers in terrestrial mollusc taxonomy likely stems from their greater access to infrastructure and resources [58,59] and saturated opportunities for local taxonomic research [12,60]. While the Global North’s surplus of taxonomic infrastructure and expertise [61,62] and well-documented biodiversity [63] drive taxonomists to study species-rich regions in the Global South, this ‘spillover’ research often excludes resident taxonomists, as evidenced by nearly 90% of parachute discoveries occurring in the Global South. Such exclusion risks facilitating poor taxonomic practices (e.g. over-splitting species) by disregarding local biodiversity expertise [64,65]. Only 75% of post-2000 species binomials in *MolluscaBase* [44] maintain accepted status, reflecting nomenclatural instability in recent descriptions. The systemic undervaluation of local knowledge extends to type-specimen collectors. Fieldwork personnel in the Global South have 1.4 times higher odds of being included as authors of domestic discoveries than Global North counterparts, whereas in parachute research, local collectors in the Global North have 1.4 times greater authorship odds than in the Global South (figure 4a), indicating that foreign researchers less frequently marginalize Global North contributions. These recognition disparities can be attributed to the Global North’s advantages in English proficiency, which facilitates communication and authorship inclusion [66–68], although ethical [69] issues and systemic over-recognition of research efforts from the Global North also play a role [57,70]. This pattern creates a paradox where northern contributions are disproportionately credited in parachute research while remaining underrepresented in domestic descriptions.

Despite evidence showing that international collaborations yield broader impact [71,72], species descriptions from the Global North show 68% lower odds of originating from mixed research collaboration than those from the Global South (electronic supplementary material, figure S1). The better infrastructure and resource access of Global North researchers [12,59] helps to improve their autonomy, which is supported by their predominance in domestic descriptions and more frequent use of advanced analytical tools (figure 4b–c). The strong correlation between GDP per capita and parachute discovery output (figure 3) further underscores the Global North’s disproportionate access to funding advantages, contrasting with the limited opportunities in the Global South. Per-country GDP influences multiple dimensions of scientific capacity, including the prevalence of top-publishing authors [61,73], overall scientific output [12,24,74], biodiversity data availability [56,59] and even the speed of species description [75,76]. These patterns reveal that biodiversity knowledge gaps and inclusivity challenges share common systemic roots. While technological democratization [38] appears to have mitigated GDP-based disparities in tool access for mollusc taxonomy, with Global South researchers showing comparable usage of internal anatomy tools in domestic research, this progress has not extended to parachute research. Species discoveries excluding resident researchers from the Global South (approx. 90% led by Global North researchers) exhibited reduced taxonomic rigour compared to those involving local experts, showing that tool access alone cannot compensate for the exclusion of local expertise [62].

Another key finding is that the higher prevalence of taxonomic revisions in species descriptions led by the Global North (89% of all revisionary studies). These revisions typically involve examining numerous specimens across multiple museum collections, often leading to the rediscovery of long-neglected species [46,75] or the synonymization of previously described taxa through modern analytical techniques [77]. This pattern aligns with recent evidence of a latitudinal taxonomy gradient, wherein temperate regions exhibit higher rates of synonymy across documented biodiversity [63,78]. The greater frequency of taxonomic revisions in wealthier nations helps explain this temperate bias in synonym prevalence, as increased research capacity facilitates more comprehensive reassessments of documented species. Since less comprehensive species descriptions are more likely to be invalidated in the future, there may be greater taxonomic instability among species described in the Global South [35,70,79]. This potential instability is not merely a nomenclatural issue; it carries practical consequences, such as difficulties in reliably identifying species for conservation assessments and the duplication of research efforts over time. Indeed, integrative species delimitation has been shown to outperform those based on single-evidence line in freshwater gastropods [80]. Addressing these issues requires capacity-building initiatives that extend beyond training researchers from the Global South, potentially in Global North institutions. Investments in infrastructure and technology transfer to institutions in the Global South are essential for achieving sustainable and autonomous research.

Although we apply the ‘North–South’ division as geopolitical proxy, we recognize its limitations in capturing national contexts and the specific capacities of research partners [81]. While country-based authorship attribution remains widely used in science [18,24,27,82] and effectively captures local constraints, this approach disregards researcher nationality—meaning parachute discoveries attribute to the Global North may actually involve Global South researchers working abroad. This could inflate parachute science estimates while simultaneously reflecting the ‘brain drain’ phenomenon [81]. Far from mitigating inequities, the diaspora of Global South expertise often exacerbates them, as emigrant researchers frequently encounter systemic barriers to career advancement [83] compounded by factors such as race, ethnicity, gender, cultural background or other socioeconomic elements [84,85]. Our assessment may have underestimated domestic discoveries from the Global South due to the underrepresentation of non-English and regional journals in our dataset—a consequence of the Global South’s limited scientific visibility. Ultimately, this is also a reflection of the lower outreach of scientific outputs from the Global South. While these limitations do not invalidate our core findings, they identify avenues for future research.

A better understanding of how geopolitical factors affect species discovery is crucial for addressing historical inequalities in investment and directing more effective strategies for cataloguing life on Earth. Global North institutions should prioritize sustainable partnerships over extractive collaborations (e.g. mandatory shared leadership roles), transparent funding (e.g. directing resources toward infrastructure for digitizing collections and establishment of molecular labs) and technology transfer (e.g. training programmes that empower local researchers), as exemplified by initiatives like Canada’s NSERC equity plans [86]. Equitable co-authorship practices, including prioritizing first-authorship for resident researchers when their fieldwork or local expertise is central, alongside long-term data sharing [26]. The neocolonial practices and geopolitical influence observed in taxonomic descriptions of molluscs illustrate the persistent challenges faced by the Global South. To advance biodiversity science, inclusivity strategies must address not only authorship parity but also accessibility to analytical tools and resources (e.g. integrating molecular tools and revisionary taxonomy) to ensure comprehensive taxonomic studies valuing local knowledge. The benefits of plural and inclusive science may go beyond equitable collaborations [23] and contribute to closing global biodiversity knowledge gaps more quickly, especially in tropical areas [9,87]. We hope these findings will contribute to paradigm shifts that strengthen global conservation efforts and improve our understanding of biodiversity worldwide.

## Data Availability

The R script and raw dataset supporting the results of this study are available from the Zenodo Digital Repository [88]. Supplementary material is available online [89].

## Authors' Notes

Our perspective on this subject is not without bias, as shown by the lack of gender diversity and the sole representation of Brazilian researchers in this work. However, we are united in our belief that science must prioritise inclusivity and ensure fair recognition for scientists from the Global South through concrete actions.
